# Gut Dysbiosis and Abnormal Bile Acid Metabolism in Colitis-Associated Cancer

**DOI:** 10.1155/2021/6645970

**Published:** 2021-02-24

**Authors:** Li Liu, Min Yang, Wenxiao Dong, Tianyu Liu, Xueli Song, Yu Gu, Sinan Wang, Yi Liu, Zaripa Abla, Xiaoming Qiao, Wentian Liu, Kui Jiang, Bangmao Wang, Jie Zhang, Hailong Cao

**Affiliations:** ^1^Department of Gastroenterology and Hepatology, General Hospital, Tianjin Medical University, Tianjin, China; ^2^Tianjin Children's Hospital, Tianjin, China; ^3^Department of Gastroenterology and Hepatology, Hotan District People's Hospital, Xinjiang Uygur Autonomous Region, Xinjiang, China; ^4^Department of Gastroenterology and Hepatology, Tianjin Third Central Hospital, Tianjin, China

## Abstract

**Background:**

Patients with prolonged inflammatory bowel disease (IBD) can develop into colorectal cancer (CRC), also called colitis-associated cancer (CAC). Studies have shown the association between gut dysbiosis, abnormal bile acid metabolism, and inflammation process. Here, we aimed to investigate these two factors in the CAC model.

**Methods:**

C57BL/6 mice were randomly allocated to two groups: azoxymethane/dextran sodium sulfate (AOM/DSS) and control. The AOM/DSS group received AOM injection followed by DSS drinking water. Intestinal inflammation, mucosal barrier, and bile acid receptors were determined by real-time PCR and immunohistochemistry. Fecal microbiome and bile acids were detected via 16S rRNA sequencing and liquid chromatography-mass spectrometry.

**Results:**

The AOM/DSS group exhibited severe mucosal barrier impairment, inflammatory response, and tumor formation. In the CAC model, the richness and biodiversity of gut microbiota were decreased, along with significant alteration of composition. The abundance of pathogens was increased, while the short-chain fatty acids producing bacteria were reduced. Interestingly, *Clostridium XlV* and *Lactobacillus*, which might be involved in the bile acid deconjugation, transformation, and desulfation, were significantly decreased. Accordingly, fecal bile acids were decreased, accompanied by reduced transformation of primary to secondary bile acids. Given bile acid receptors, the ileum farnesoid X receptor-fibroblast growth factor 15 (FXR-FGF15) axis was downregulated, while Takeda G-protein receptor 5 (TGR5) was overexpressed in colonic tumor tissues.

**Conclusion:**

Gut dysbiosis might alter the metabolism of bile acids and promote CAC, which would provide a potential preventive strategy of CAC by regulating gut microbiota and bile acid metabolism.

## 1. Introduction

Colorectal cancer (CRC) is one of the most common cancers worldwide [1] and can be identified as sporadic, hereditary CRC, or colitis-associated cancer (CAC) [2, 3]. Patients with long-term inflammatory bowel disease (IBD), especially ulcerative colitis, can develop into intestinal cancer, known as CAC. The risks of developing CAC in IBD patients were 2% by 10-year intervals, 8% by 20 years, and 18% by 30 years, as shown in a meta-analysis [4]. Specific factors of IBD patients can increase the prevalence of CAC, such as extensive mucosal involvement, the severity and duration of the disease, family history, primary sclerotizing cholangitis, and therapeutic effect of the disease [5, 6]. Factors involving CAC development include immune response, epigenetic modification, intestinal inflammatory response, and gut dysbiosis [7, 8].

Previous studies have indicated the relevance between altered gut microbiota and risk of gastrointestinal diseases (such as IBD, CRC, and irritable bowel syndrome) [6, 9, 10]. Gut microbiota maintains host health by participating in immune modulation and host metabolism [8]. Moreover, the presence of gut microbiota plays a crucial role in bile acid metabolism [9, 11]. Bile acids are synthesized by classical and alternative pathways in hepatocytes. The bile acids conjugated to either taurine or glycine are finally transported to the intestine. Bile salt hydrolase (BSH) containing bacteria can convert bile acids from conjugated to unconjugated, and bacteria that possess 7*α*-dehydroxylation activity can make primary bile acids transform into secondary bile acids. In the distal ileum, almost 95% of bile acids are returned to the liver [12, 13]. Physiologically, bile acids can regulate extensive metabolic and immune-related activities including glucose, lipid, and energy metabolism [14, 15]. Nevertheless, excessive bile acids in the intestine especially secondary bile acids have the capability of promoting CRC. Previous literature has shown that gut dysbiosis and bile acid metabolism disorder can promote CRC [10, 16]. However, the role of these two in the CAC progression is not fully understood.

Bile acids also exert metabolic effects by activating bile acid receptors, mainly the nuclear receptor farnesoid X receptor (FXR) and G-protein coupled receptor (TGR5) [17]. FXR, mainly expressed in the liver, kidney, and terminal ileum, has a significant influence on bile acids, liquid, and glucose metabolic homeostasis [15, 18–20]. Activation of intestinal FXR is responsible for bile acid reabsorption through the portal vein and limits the uptake of bile acids in the enterocytes [21, 22]. TGR5, highly expressed in the ileum, colon, and gallbladder, can regulate the energy homeostasis and bile acids, liquid and glucose metabolism, cell proliferation, and apoptosis and immune responses [23, 24]. It has shown that the TGR5 is highly expressed in esophageal and gastric adenocarcinoma [25, 26]; however, the role of TGR5 in CAC remains unclear.

We hypothesized that gut microbiota and bile acid metabolism could be involved in CAC development, and we chose the AOM/DSS model in the present study. Our results revealed gut dysbiosis during tumorigenesis, accompanied by abnormal bile acid metabolism. In addition, FXR and TGR5, the two main bile acid receptors, were also involved in CAC. These results provide a better understanding of CAC, suggesting that the regulation of gut microbiota and bile acids might be a guiding therapeutic strategy for CAC.

## 2. Materials and Methods

### 2.1. Animals and Induction of CAC

**Figure 1:**
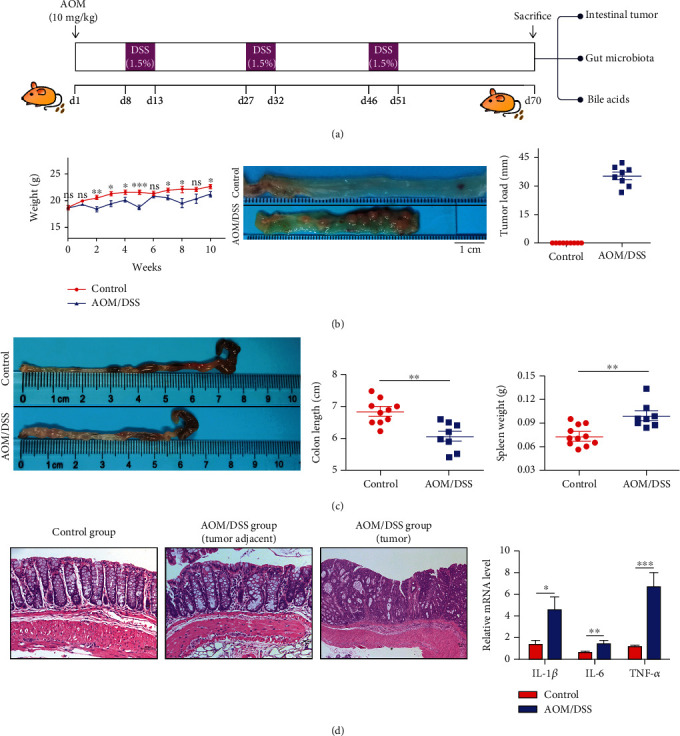
Tumor formation and severe inflammatory response in the colon of mice treated with AOM/DSS. (a) Mice received intraperitoneal injection of 10 mg/kg AOM on day 1 and followed by three circles of 1.5% DSS drinking water in the AOM/DSS group. And all mice were killed on day 70. (b) Body weight, colon lumen appearance image, and tumor load of the two groups. (c) AOM/DSS treatment shortened the colon length and increased the weight of the spleen. (d) H&E staining revealed colon tumor formation and inflammatory cell infiltration in the AOM/DSS group. Real-time PCR showed the increased levels of several inflammatory cytokines (IL-1*β*, IL-6, and TNF-*α*) in the colon. ^∗^*p* < 0.05, ^∗∗^*p* < 0.01, and ^∗∗∗^*p* < 0.001. *n* = 8-10.

In the present study, we chose the AOM/DSS-induced CAC model, which had the advantages of reproducibility, simplicity, affordability, and mainly invading the colon, similar to human sporadic CRC [27]. Twenty female C57BL/6 mice aged 7 weeks were obtained from Beijing Huafukang Bioscience Co. Inc. (Beijing, China) and acclimatized 1 week before the experiment. They were randomly divided into the AOM/DSS and control groups with 10 mice, respectively. All mice were maintained in a specific pathogen-free (SPF) condition with the 12 : 12 light-dark cycle. The mice were fed a diet of AIN-93M rodents and free to eat and drink. According to our previous study and literatures [28–30], intraperitoneal injection of 10 mg/kg azoxymethane (AOM) (Sigma, USA) was applied to the AOM/DSS group, while the control group was intraperitoneally injected with sterile isotonic saline on day 1. After seven days, the AOM/DSS group was given 1.5% dextran sodium sulfate (DSS) (MP Biomedicals, USA) in drinking water on days 8–13, 27–32, and 46–51, and each cycle of DSS treatment was followed by 14-day drinking water. Mice were euthanized by CO_2_ asphyxiation on day 70 (Figure 1(a)). Animal experiments were performed following the experimental regulations of the Animal Ethics Committee.

### 2.2. Tissue and Feces Collection

All mice were observed every day and weighed weekly. The general condition and defecation of the mice were recorded during AOM/DSS treatment. On days 0 and 70, each mouse was individually housed in a clean cage for two hours to collect feces. Then, mice were sacrificed with measurement of colon length and spleen weight. The intestine was washed with ice PBS and dissected longitudinally. The location, size, and numbers of intestinal tumors were observed. Tumor load refers to the sum of the tumor diameters of each mouse. Intestinal tissues (ileum and colon) were stored at -80°C for subsequent study. The colon was rolled and embedded in paraffin for further pathological and immunohistochemistry analysis.

### 2.3. Pathology and Immunohistochemistry

Colon tissue was cut into sections (5 *μ*m), and then, hematoxylin and eosin (H&E) staining was applied to colon sections for assessment of tumor and inflammatory cell infiltration. In addition, colon sections were deparaffinized and rehydrated for immunohistochemistry to detect the expression of TGR5. Slides were incubated with rabbit monoclonal TGR5 antibody (1 : 100, Abcam, MA, USA) at 4°C overnight, followed by corresponding secondary antibody. Then, the sections were treated with horseradish peroxidase- (HRP-) streptavidin solution. Finally, 3,3′-diaminobenzidine was applied for counterstaining and further observation. At least five areas from each single section were observed under light microscope DM5000B (Leika, Germany).

### 2.4. Immunofluorescent Staining

Colon sections were incubated with primary antibody ZO-1 (Abcam, MA, USA) in a humid chamber for 12 h at 4°C. Subsequently, after washing with PBS slightly, the fluorescently conjugated secondary antibody was applied. And this incubation process lasted for 1 h at room temperature. After DAPI reaction and seal, the slides were observed with a fluorescence microscope, and then, we obtained DAPI and FITC images of a unified area.

### 2.5. Real-time PCR Analysis

Total RNA was extracted from the intestinal tissues (ileum and colon) by a RNeasy mini kit. Reverse transcription of cDNA was performed with the TIANScript RT Kit. Real-time PCR analysis was performed by the Applied Biosystems StepOnePlus™ Real-time PCR System. Each run consisted of 95°C for 10 min, followed by 40 cycles of 95°C for 15 s and 60°C for 60 s, and then 95°C for 15 s, 60°C for 60 s, and 95°C for 15 s in a 20 ml volume. The levels of mRNA were analyzed by the *ΔΔ*Ct method. The oligonucleotide primer sequences are listed in Table 1.

### 2.6. Gut Microbiota Analysis

The 16S rRNA sequencing was performed using the Illumina HiSeq PE250. DNA was extracted with the QIAamp DNA Stool Mini kit (Qiagen, Germany). Then, the primer F341 (5′-ACTCCTACGGGRSGCAGCAG-3′) and R806 (5′-GGACTACVVGGGTATCTAATC-3′) were designed for 16S rRNA gene (V3-V4 region) amplification. The sequences from samples of the two groups were clustered to generate operational taxonomic units (OTUs) at the 97% identity using Usearch. The representative sequence of each OTU was classified using the RDP database. Alpha diversity and beta diversity were performed using QIIME.

### 2.7. Measurement of Bile Acids in Feces

The liquid chromatography-mass spectrometry (LCMS) method was applied to measure fecal bile acid concentration. As reference standards, cholic acid (CA), chenodeoxycholic acid (CDCA), and lithocholic acid (LCA) were purchased from Aladdin, with *α*-muricholic acid (*α*-MCA) and *β*-muricholic acid (*β*-MCA) from Toronto Research Chemicals, and deoxycholic acid (DCA) from Sigma. And they were added to fecal samples for preliminary measurement by an external standard method. Each fecal sample was suspended in 5 ml of chromatographic ethanol and then ultrasonically extracted for 60 min at 30°C. After 10 minutes of centrifugation (10,000 rpm, 4°C), the supernatant (4 ml) was aspirated and dried under nitrogen. The samples were redissolved with methanol and went through a 0.22 *μ*m filter. Finally, bile acids were analyzed using the Agilent 1260 Series liquid chromatograph combined with a 6120B mass spectrometer. The concentrations of bile acids were determined based on the peak areas [31, 32].

### 2.8. Statistical Analysis

The data were described as the mean ± SEM. Differences between the two groups were determined by Student's *t*-test. GraphPad Prism 5.01 and SPSS 22.0 were applied for data analysis. *P* < 0.05 was considered significant.

## 3. Results

### 3.1. General Characteristics of CAC Mouse Model

Mice in the control group grew well, while two mice in the AOM/DSS group died while receiving DSS. Mice in the AOM/DSS group showed noticeable weight loss, accompanied by hematochezia during each cycle of DSS treatment. At 10 weeks, tumor load of the AOM/DSS group was 35.41 ± 1.901 mm, characterized by shortened colon length 6.06 ± 0.158*vs.*6.83 ± 0.125 cm and increased spleen weight 0.10 ± 0.006*vs.*0.07 ± 0.004 g (Figures 1(b) and 1(c). H&E staining revealed significant inflammatory cell infiltration and intramucosal tumor in the colon of the AOM/DSS group, and the mRNA levels of inflammatory cytokines (IL-1*β*, IL-6, and TNF-*α*) were significantly increased (Figure 1(d)).

### 3.2. Intestinal Barrier Disruption and Apoptosis Inhibition after AOM/DSS Treatment

**Figure 2:**
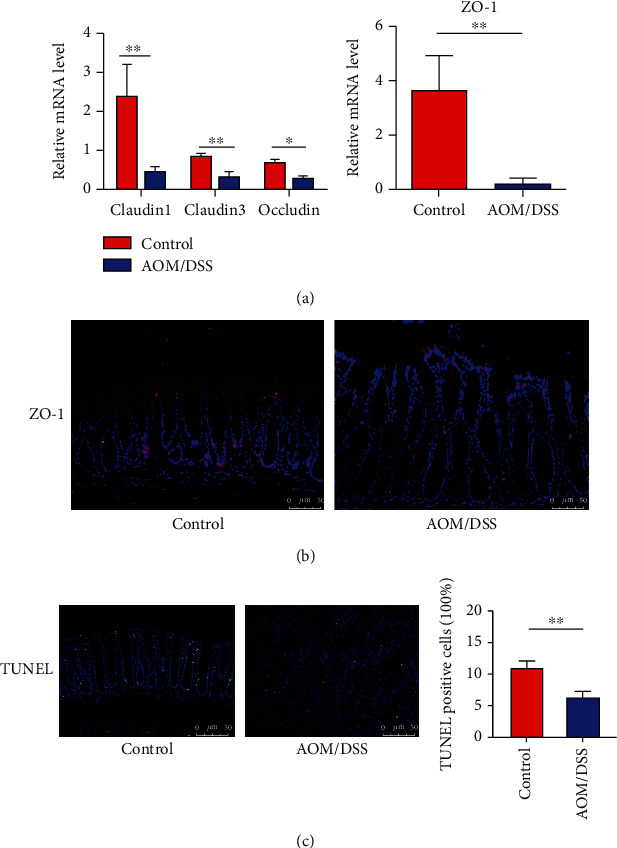
Intestinal barrier was disrupted in the CAC model. (a) The mRNA level of Occludin, Claudin1, Claudin3, and ZO-1 was reduced in the colon after AOM/DSS treatment. (b) Immunofluorescent staining for ZO-1 in colon tissues of the control and AOM/DSS group. (c) Colon sections from the two groups were stained with TUNEL. Data were quantified as the mean percentage of positive-stained cells in five randomly selected fields in each sample. Scale bars, 50 *μ*m. ^∗^*p* < 0.05, ^∗∗^*p* < 0.01. *n* = 8-10.

The intestinal barrier exists as an effective defense system to maintain homeostasis of the host. Tight junctions including ZO-1, Claudins, and Occludin are critical in preventing the penetration of pathogenic microorganisms. The mRNA expression of ZO-1, Occludin, Claudin1, and Claudin3 was significantly reduced in the colon of the AOM/DSS group (Figure 2(a)), indicating that the mucosal barrier was disrupted in the development of CAC. Additionally, the expression of ZO-1 in immunofluorescence was decreased after AOM/DSS treatment (Figure 2(b)). The AOM/DSS group showed significantly decreased apoptotic cells than the control group (6.24 ± 0.82*vs.*10.95 ± 1.08, *P* < 0.01, Figure 2(c)), hinting the inhibition of cell apoptosis in the CAC model.

### 3.3. Decreased Gut Microbiota Diversity in the Development of CAC

**Figure 3:**
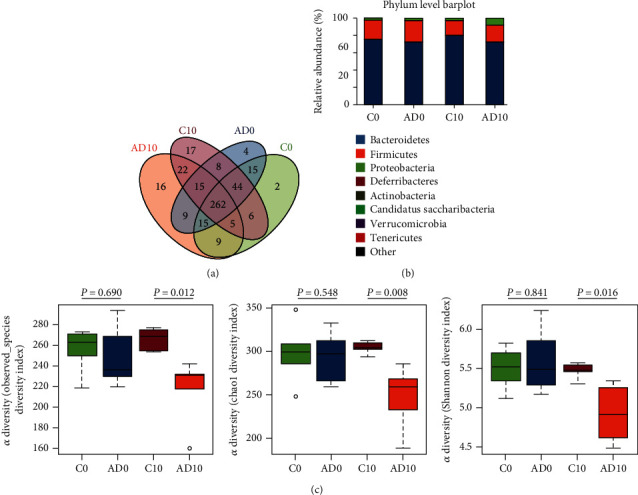
The gut microbiota composition at 0 and 10 weeks. (a) Venn diagram in the control and AOM/DSS group. (b) The abundance of *Bacteroidetes* decreased in the AOM/DSS group at 10 weeks, and the *Firmicutes* increased. (c) The *α*-diversity (observed species, chao1, and Shannon) showed no significant distinction between the two groups at 0 weeks, but it was dramatically reduced in the AOM/DSS group compared with the control group at 10 weeks. C0 and C10: control group at 0 and 10 weeks. AD0 and AD10: AOM/DSS group at 0 and 10 weeks. *n* = 5 in each group.

A total of 372 and 353 OTUs were detected in the AOM/DSS group, while the control group was 358 and 379 OTUs at 0 and 10 weeks (Figure 3(a)). At the phylum level, compared with the control group, the abundance of *Firmicutes* increased (17.3% *vs.* 19.8%) in the AOM/DSS group at 10 weeks, and the *Bacteroidetes* decreased (79.7% *vs.* 72.1%, Figure 3(b)). Since there was no statistical difference in *α*-diversity between the two groups at 0 weeks (*P* > 0.05), the observed species, chao1, and Shannon index were significantly reduced in the AOM/DSS group after 10 weeks (*P* < 0.05), suggesting a decreased of gut microbiota richness and diversity in the CAC model (Figure 3(c)).

### 3.4. Alteration of Gut Microbiota Composition in the Development of CAC

**Figure 4:**
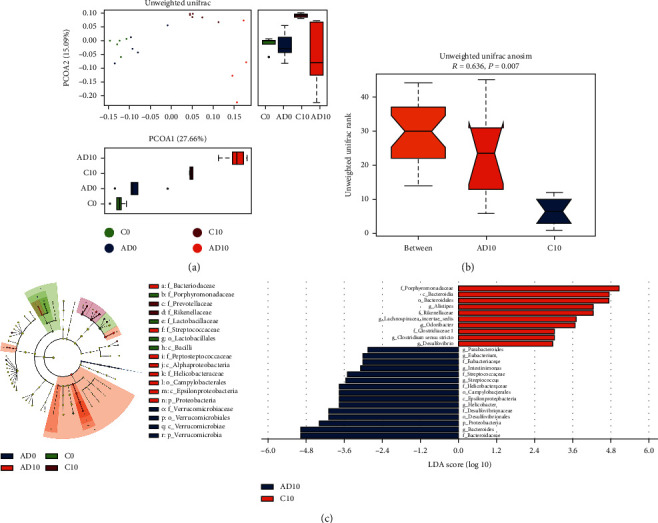
The alteration of gut microbiota during colitis-associated cancer development. (a) The PCoA plot showed a significant distinction in microbial composition among the two groups at 10 weeks. (b) Unweighted Unifrac Anosim analysis suggested a reasonable grouping after AOM/DSS treatment. (c) The LefSe analysis listed bacteria with significant differences at different levels in each group. C0 and C10: control group at 0 and 10 weeks. AD0 and AD10: AOM/DSS group at 0 and 10 weeks. *n* = 5 in each group.

The principal coordinate analysis (PCoA) plot showed that the microbial composition among the two groups was similar at 0 weeks and especially distinct after 10 weeks (Figure 4(a)). Unweighted Unifrac Anosim analysis showed that the *R* value was 0.636 and the *P* value was 0.007, which indicated pronounced differences in species diversity between the two groups at 10 weeks (Figure 4(b)). The LefSe analysis was applied to evaluate the differential abundant species of the two groups at different levels (Figure 4(c)). The fecal microbiota results at week 10 showed a high abundance of the family *Bacteroidaceae*, *Eubacteriaceae*, and *Helicobacteraceae* in the AOM/DSS group and low abundance of *Clostridiaceae 1*, *Porphyromonadaceae*, and *Rikenellaceae*. At the genus level, the abundance of pathogens *Helicobacter* and *Streptococcus* was increased in the CAC model, and the short-chain fatty acids (SCFAs) producing bacteria including *Alistipes*, *Lachnospiracea_incertae_sedis*, and *Odoribacter* were decreased. Interestingly, the abundance of *Clostridium XlV* and *Lactobacillus*, which might be engaged in the metabolic process of bile acids, was decreased in the AOM/DSS group.

### 3.5. Fecal Bile Acid Profile in the CAC Model

**Figure 5:**
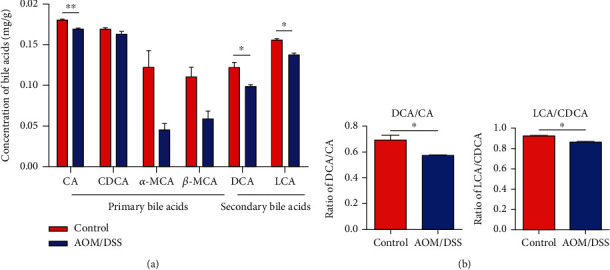
The bile acid profile alteration in feces after AOM/DSS treatment. (a) Levels of fecal bile acids including CA, DCA, and LCA were reduced in the AOM/DSS group. (b) The conversion of primary bile acids to secondary bile acids was reduced in the AOM/DSS group, as evidenced by a decreased ratio of DCA/CA and LCA/CDCA. ^∗^*p* < 0.05, ^∗∗^*p* < 0.01. *n* = 5 in each group.

To investigate bile acid metabolism during CAC development, the concentration in feces was tested by LCMS. After AOM/DSS treatment, levels of CA, DCA, and LCA in the feces were significantly reduced (*P* < 0.05, Figure 5(a)). Importantly, the ratio of DCA/CA and LCA/CDCA also decreased in the AOM/DSS group, indicating an impaired conversion from primary bile acids to secondary bile acids (Figure 5(b)). As previously mentioned, the abundance of *Clostridium XlV* and *Lactobacillus*, which were associated with bile acid metabolism, was reduced in the AOM/DSS group. Thus, the ability to bile acid deconjugation, transformation, and desulfation might be impaired after AOM/DSS treatment.

### 3.6. Bile Acid Receptors FXR and TGR5 in CAC Development

**Figure 6:**
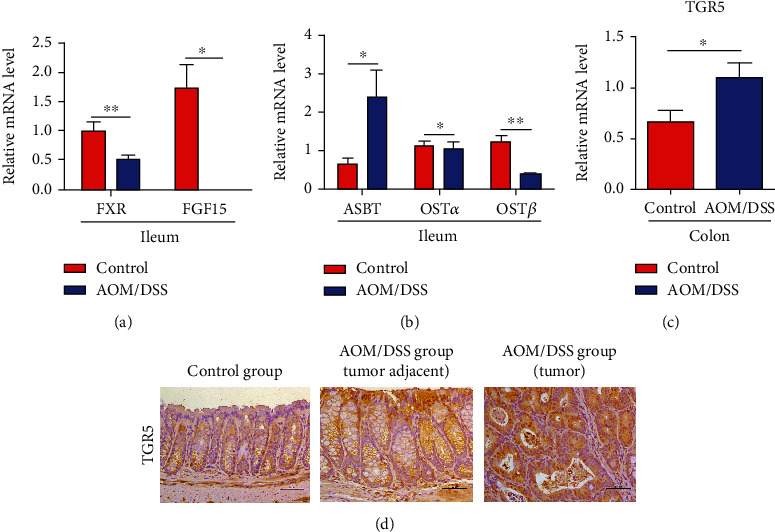
The expression of bile acid receptors in the colitis-associated cancer model. (a) The mRNA expression of ileum FXR and FGF15 was reduced in the AOM/DSS group. (b) Real-time PCR showed an increased expression of ASBT and a decreased level of OST*α* and OST*β* in the ileum of the AOM/DSS group. (c) The mRNA expression of colon TGR5 was elevated in the tumor of the AOM/DSS group by Real-time PCR. (d) Immunohistochemical staining showed higher expression of TGR5 in mice with AOM/DSS treatment than in the control group. ASBT: apical sodium-dependent bile acid transporter. OST*α* and OST*β*: organic solute transporter subunit *α* and *β*. Scale bars: 50 *μ*m. ^∗^*p* < 0.05, ^∗∗^*p* < 0.01. *n* = 8-10.

Bile acid receptors FXR and TGR5 can be activated by bile acids. Real-time PCR showed decreased levels of ileum FXR and fibroblast growth factor 15 (FGF15) in the AOM/DSS group (Figure 6(a)). Consistent with this, the expression of organic solute transporter subunits *α* and *β* (OST*α* and OST*β*) was reduced, while the apical sodium-dependent bile acid transporter (ASBT) was highly expressed, which led to the accumulation of bile acids in enterocytes and limited the return to the liver (Figure 6(b)). Moreover, our results also showed that TGR5 mRNA expression was higher in the colon of mice after AOM/DSS treatment than in the control group (Figure 6(c)). Simultaneously, immunohistochemical staining confirmed a high expression of TGR5 in the AOM/DSS group (Figure 6(d)).

## 4. Discussion

**Figure 7:**
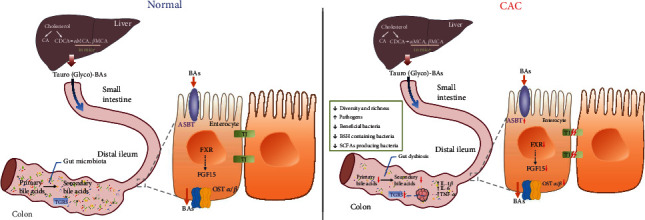
Gut dysbiosis and abnormal bile acid metabolism in colitis-associated cancer. Synthesized in the liver, bile acids are transported to the intestine in the form of conjugated bile acids. In the distal ileum, most of them are reabsorbed and conveyed to the liver in the presence of the FXR-FGF15 axis and bile acid transport receptors. In the colon, the gut microbiota promotes bile acid deconjugation and the conversion of primary bile acids to secondary bile acids. After AOM/DSS treatment, the BSH containing bacteria is reduced. Thus, dysbiosis inhibits bile acid metabolism and induces decreased secondary bile acids. Considering the bile acid receptors, the expression of FXR-FGF15 axis, OST*α*, and OST*β* is decreased, while ASBT is increased, which limits the reabsorption of bile acids and leads to the accumulation of bile acids in enterocytes. The colon of the CAC model shows a severe inflammatory response, disrupted barrier function, and elevated expression of TGR5 in tumor tissues. BAs: bile acids; ASBT: apical sodium-dependent bile acid transporter; FXR: farnesoid X receptor; FGF15: fibroblast growth factor 15; TGR5: G-protein coupled receptor; OST*α* and OST*β*: organic solute transporter subunit *α* and *β*; BSH: bile salt hydrolase; CAC: colitis-associated cancer; TJ: tight junction.

It has been pointed out that 18% of IBD patients may develop CRC 30 years after colitis is diagnosed, known as CAC [4]. Substantial evidence has demonstrated that gut dysbiosis and abnormal bile acid metabolism exist in many diseases such as CRC, nonalcoholic fatty liver disease, and diabetes. Our previous studies have reported that bile acid-induced dysbiosis promoted intestinal tumorigenesis in *Apc*^min/+^ mice [32, 33]. Our results in the present study showed destroyed intestinal barrier and colon tumor formation after AOM/DSS treatment. Meanwhile, the abundance of Helicobacter and Streptococcus, known as pathogens, was increased. Interestingly, the BSH containing bacteria *Clostridium XlV* and *Lactobacillus* were reduced with the decreased conversion of primary bile acids to secondary bile acids. Furthermore, the bile acid receptor FXR-FGF15 axis was downregulated. Our results suggested that gut dysbiosis inhibited the bile acid metabolism, led to the accumulation of bile acids in enterocytes, and promoted tumorigenesis in the CAC model (Figure 7). Taken together, it will provide a new insight that gut dysbiosis and abnormal bile acid metabolism play a crucial role in CAC development.

*Firmicutes* and *Bacteroidetes* are the dominant phylum bacteria in the intestine. Our data revealed the decreased abundance of *Bacteroidetes* and increased *Firmicutes* in the CAC model. At the genus level, the *Lachnospiracea_incertae_sedis*, *Alistipes*, and *Odoribacter*, known as the short-chain fatty acids (SCFAs) producing bacteria, were decreased after AOM/DSS treatment. As a vital source of energy, SCFAs can provide energy for colonic epithelial cells. Simultaneously, they are responsible for epithelial barrier enhancement and gastrointestinal immunological regulation [34]. Therefore, the dysbiosis impaired the production and protective effect of SCFAs. Besides, previous studies had shown a reduced abundance of *Alistipes* in IBD patients [35], and *Alistipes* was reported to play a role in alleviating colitis [36]. Thus, in the CAC model, the reduction of *Alistipes* might diminish its protective effect. On the contrary, the level of pathogens such as *Helicobacter* and *Streptococcus* increased. In addition, *Parabacteroides* and *Bacteroides* have been reported to have higher levels in CRC patients [37], which also remarkably increased in the CAC model in our study, suggesting that *Parabacteroides* and *Bacteroides* are involved in intestinal tumorigenesis of CAC. The above results indicated a pronounced increase in pathogens and reduction in beneficial bacteria during CAC progression.

We also observed a decreased output of fecal bile acids after AOM/DSS treatment. The ratios of DCA/CA and LCA/CDCA, which represent the conversion of primary bile acids to secondary bile acids, were also reduced. Secondary bile acids have been reported to have anti-inflammatory effects. For example, DCA can inhibit TNF-*α* production [38] and LCA can downregulate NF-*κ*B activity in colon cells [39]. Additionally, DCA and LCA restrained the IL-8 secretion and exerted anti-inflammatory effects on Caco-2 cells [40]. Alternatively, genus *Clostridium XlV* and *Lactobacillus* were reduced in CAC. It is well known that *Clostridium XlV* and *Lactobacillus* have bile salt hydrolase (BSH) activity, and *Clostridium XlV* also possesses 7*α*-dehydroxylation and bile acid sulfatase activity [12, 13]. The reduction may account for the impaired ability of bile acid deconjugation, transformation, and desulfation activity in CAC. An intriguing study has similar results in IBD, which showed higher levels of fecal sulfated and conjugated bile acids in IBD patients than the healthy subjects [40]. Similarly, a recent study found the reduction of LCA and DCA and the relative overabundance of primary bile acids in IBD subjects by detecting the metabolomic profiles of stool samples [41]. Thus, the reduction of secondary bile acids might be one of the causes of CAC.

Bile acids can directly regulate gut microbiota or through the bile acid receptors FXR and TGR5 [42, 43]. Bile acids are regarded as FXR agonists, and the order of activation effect is CDCA > DCA > LCA > CA [44]. The reduction of DCA, LCA, and CA levels in our study led to the inactivation of FXR. Moreover, a decreased abundance of *Lactobacillus* caused the accumulation of conjugated bile acids, such as T-*β*-MCA, which has been reported as the FXR antagonist [45], so a high level of T-*β*-MCA may be involved in the decreased expression of FXR [46]. The downregulation of the FXR-FGF15 axis decreased the bile acid efflux transporters and affected the reabsorption of bile acids. In our study, the expression of FXR, OST*α*, and OST*β* was decreased, while ASBT was increased, which resulted in the accumulation of bile acids in the enterocytes. These data revealed that abnormal bile acid metabolism was involved in CAC development. Several studies have found that the FXR mRNA expression is inversely correlated with CRC progression, and FXR deficiency increased the tumor load in *Apc*^min/+^ mice and xenograft tumor model [47–49]. Moreover, mice lacking FXR showed disrupted intestinal epithelium integrity and an overgrowth of intestinal bacteria [50]. It has been found that FXR activation reduced intestinal inflammation and syndrome and improved intestinal mucosal barrier in the DSS-induced colitis model [51]. In esophageal and gastric adenocarcinoma, the expression of TGR5 elevates remarkably [25, 26]. Furthermore, TGR5 has been found increased in an inflammatory state of the colitis model and Crohn's disease patients [52, 53]. Moreover, TGR5 agonist has been reported to ameliorate colitis [54]. We found a higher level of TGR5 in the tumor tissues of CAC. Thus, targeting bile acid receptors FXR and TGR5 would be a promising approach against CAC.

## 5. Conclusion

These data suggested that gut dysbiosis might affect the bile acid metabolism during the development of CAC, and the reduced production of secondary bile acids with anti-inflammatory effects could promote tumorigenesis. Our study revealed a pivotal role of gut microbiota and bile acids in CAC progression, which may provide a new preventive strategy against CAC.

## Figures and Tables

**Table 1:** The oligonucleotide primer sequences used in the experiments.

Primers	Sequence
GAPDH	Forward 5′-TGTGTCCGTCGTGGATCTGA-3′ Reverse 5′-CCTGCTTCACCACCTTCTTGA-3′
TNF-*α*	Forward 5′-ACTCCAGGCGGTGCCTATG-3′ Reverse 5′-GAGCGTGGTGGCCCCT-3′
IL-1*β*	Forward 5′-GTGGCTGTGGAGAAGCTGTG-3′ Reverse 5′-GAAGGTCCACGGGAAAGACAC-3′
IL-6	Forward 5′-CCAGTTGCCTTCTTGGGACT-3′ Reverse 5′-GGTCTGTTGGGAGTGGTATCC-3′
ZO-1	Forward 5′-GGGCCATCTCAACTCCTGTA-3′ Reverse 5′-AGAAGGGCTGACGGGTAAAT-3′
Occludin	Forward 5′-CGGTACAGCAGCAATGGTAA-3′ Reverse 5′-CTCCCCACCTGTCGTGTAGT-3′
Claudin1	Forward 5′-AGACCTGGATTTGCATCTTGGTG-3′ Reverse 5′-TGCAACATAGGCAGGACAAGAGTTA-3′
Claudin3	Forward 5′-CCTGTGGATGAACTGCGTG-3′ Reverse 5′-GTAGTCCTTGCGGTCGTAG-3′
FXR	Forward 5′-GGACGGGATGAGTGTGAAG-3′ Reverse 5′-TGAACTTGAGGAAACGGGAC-3′
FGF15	Forward 5′-TGAAGACGATTGCCATCAAGG-3′ Reverse 5′-GGATCTGTACTGGTTGTAGCC-3′
ASBT	Forward 5′-AGGAATACTGTACCAAAGTGCC-3′ Reverse 5′-TTTCCAAGGCTACTGTTCGG-3′
OST*α*	Forward 5′-TGCTCACCTCCCTACTCTTC-3′ Reverse 5′-AACAAGCCTCATACCCAACC-3′
OST*β*	Forward 5′-GCTTTGGTATTTTCGTGCAGAAG-3′ Reverse 5′-GTTTCTTTGTCTTGTGGCTGC-3′
TGR5	Forward 5′-AAAGGTGTCTACGAGTGCTTC-3′ Reverse 5′-TGCATTGGCTACTGGTGTG-3′

## Data Availability

The related data underlying the findings of this research are freely available. And readers can make requests for access to these data to the corresponding author via email.
